# Seeking Alfalfa Resistance to a Rhizophagous Pest, the Clover Root Curculio (*Sitona hispidulus* F.)

**DOI:** 10.3390/insects12100906

**Published:** 2021-10-05

**Authors:** Kaitlin Rim, Jamie Crawford, Steven J. Price, Donald R. Viands, Ricardo A. Ramirez

**Affiliations:** 1 Department of Biology, Utah State University, Logan, UT 84322, USA; rimkaitlin@gmail.com; 2 Section of Plant Breeding and Genetics, School of Integrated Plant Sciences, Cornell University, Ithaca, NY 14853, USA; jln15@cornell.edu (J.C.); donald.viands@cornell.edu (D.R.V.); 3 Carbon County Extension, Utah State University, Price, UT 84501, USA; steven.price@usu.edu

**Keywords:** host-plant resistance, screening, feeding behavior, oviposition, belowground, soil, weevil

## Abstract

**Simple Summary:**

Clover root curculio (CRC) is a root feeding pest of alfalfa and clover that reduces stand life and yield. With the cancellation of soil-active insecticides in alfalfa, CRC populations and associated root damage have increased. Current CRC management practices are limited in their ability to suppress larval feeding belowground. Here, we evaluated alfalfa populations for resistance to CRC larval feeding and development belowground, and adult leaf consumption and oviposition aboveground. Divergent selection in two alfalfa cultivars in field nurseries revealed that there is genetic variability in resistance to CRC larval feeding and that significant gains in resistance from selection can occur in as few as two or three cycles of selection. While larval development was similar across the alfalfa populations tested in the lab, one alfalfa population (NY1713) displayed an overall increase in nodulation resulting in significantly lower proportions of nodules being consumed by larvae. These results provide possible candidates and soil-less method for the development and evaluation of alfalfa cultivars that may reduce the impacts of CRC root feeding and that offer an additional option for CRC management.

**Abstract:**

Since the cancellation of broad-spectrum soil-active insecticides in alfalfa (*Medicago sativa* L.) production, clover root curculio (*Sitona hispidulus* F.) (CRC) larval root damage has increased. Current CRC management practices are limited in their ability to suppress larval feeding belowground. First, we field screened developmental alfalfa populations for CRC damage. Subsequently, we developed a soil-less arena to observe nodule feeding and development (head capsule width) of larvae in the lab. This method was used to evaluate five alfalfa populations (two CRC-susceptible (control) and three CRC-resistant populations) against larvae. Further, one CRC-resistant population paired with its genetically similar susceptible population were tested against adult leaf consumption and oviposition in the greenhouse. Field screening revealed that the alfalfa populations selected for little or no larval root feeding damage were more resistant to CRC larval feeding than their corresponding unselected cultivars and significantly more resistant than populations selected for susceptibility. The development of a soil-less arena provided a useful method for evaluation of root-larva interactions. Although larval development was similar across susceptible and resistant alfalfa populations, one CRC-resistant population (NY1713) displayed overall increased nodulation and, thus, had a significantly lower proportion of nodules consumed by larvae. Adult feeding and oviposition aboveground were similar across all populations tested. These results provide possible candidates and screening method for the development and evaluation of alfalfa cultivars that may reduce the impacts of larval feeding and that offer an additional option for CRC management.

## 1. Introduction

Clover root curculio (CRC) [*Sitona hispidulus* F. (Coleoptera: Curculionidae)] is oligophagous on Fabacea but is primarily an alfalfa and clover (Fabales: Fabaceae) pest that negatively affects alfalfa plant health and yield. CRC is univoltine and widely distributed throughout the continental United States, from as far north as Alaska and south to Mexico [1]. Adults feed aboveground on leaves, yet typically do not cause economic losses [1]. Conversely, immature CRC feed on roots belowground and are recognized as the damaging stage [1]. Larval feeding can result in reduced stand life and plant density, stunting, yield loss, and decreased plant overwintering survival (e.g., [2,3,4]). Furthermore, larval feeding damage increases plant susceptibility to *Fusarium* (Hypocreales: Nectriaceae) infections which can exacerbate these crop health issues [5,6,7]. Since the cancellation of broad-spectrum soil-active insecticides (e.g., carbofuran) and fumigants commonly used against pests like alfalfa weevil [*Hypera postica* Gyllenhal (Coleoptera: Curculionidae)] [8], there have been notable increases in CRC populations and associated root damage [9]. Furthermore, broad-spectrum insecticides (e.g., pyrethroids) registered for CRC adult management may have detrimental non-target effects on beneficial predators and pollinators [10], and current management practices are limited in their ability to suppress cryptic CRC larvae feeding belowground [1]. Thus, developing integrated pest management tactics is critically needed.

The development of tolerant or resistant host plants has been integral to successful integrated pest management programs in cropping systems. Over 100 alfalfa cultivars are commercially available with varying resistance to a multitude of alfalfa pests [11]. However, none of these commercial alfalfa cultivars are resistant to chewing insects such as alfalfa weevil or CRC, in part due to the high genetic variability in alfalfa [12], and also considering that mechanisms underlying alfalfa resistance to chewing insects (e.g., saponins) may potentially interfere with palatability (e.g., bloat in livestock [13]).

Historic evaluations for CRC resistance identified potentially resistant cultivars (e.g., Lahontan), but have yet to identify resistance mechanisms [14,15]. However, research on congeners may shed some light on host plant characteristics that may confer resistance to CRC. For instance, as leaf thickness increased for pea plants, herbivory from adult *Sitona lineatus* L. (Coleoptera: Curculionidae) decreased [16]. Additionally, sweet clover root disks impregnated with nitrates, arabinose, ascorbic acid, glucuronic acid, or mannitol, all isolated from healthy plants, deterred *Sitona cylindricollis* Fåhraeus (Coleoptera: Curculionidae) feeding [17]. The Cornell Forage Breeding and Entomology Programs (Ithaca, NY, USA) have given additional promise in combating alfalfa chewing insects with the development and release of an alfalfa snout beetle [*Otiorhychus lingustici* L. (Coleoptera: Curculionidae)] resistant cultivar (Seedway 9558 SBR) [18]. This weevil causes significant root damage to alfalfa in northern New York, similar to damage caused by CRC larvae [19]. Moreover, development of CRC-resistant alfalfa populations has been progressing [20]. Some of the challenges in these evaluations include successfully rearing CRC for manipulative studies, the patchy distribution of CRC populations in the field, the large undertaking in effort to sample and collect larvae from soil, and the lack of methods to quickly screen and evaluate the larval interactions with roots and nodules. 

Considering the ongoing selection and development of insect resistant alfalfa populations, we field screened 363 half-sib families from three previously selected populations selecting the most vigorous plants with the least amount of larval feeding damage. We hypothesized that additional cycles of selection within these populations would continue to improve CRC resistance given that the Cornell Forage Breeding Project previously developed and released an alfalfa cultivar resistant to another root weevil (i.e., alfalfa snout beetle) and that initial field screenings of experimental CRC-resistant populations showed higher crown and root biomass and lower root damage ratings [20]. As a second aim, we looked to develop a soil-less method to investigate direct CRC larval feeding and to use this arena as an approach for belowground screening of plant roots with insect larvae. Taking this approach, we evaluated five candidate alfalfa populations (resistant and susceptible to CRC) from our field screening to directly investigate larval nodule feeding and development of early instar larvae by measuring head capsule width. Taking the single most resistant population to larval feeding and its paired susceptible population from divergent selection, we then evaluated leaf consumption by adult CRC and oviposition aboveground. We hypothesized that CRC-resistant populations would exhibit reduced adult feeding and oviposition aboveground, and decreased nodule feeding and CRC larval development belowground. These objectives provide methods for the selection, screening, and direct evaluation of CRC belowground in the development of alfalfa less impacted by CRC and additional options for CRC management in alfalfa. 

## 2. Materials and Methods

### 2.1. Field Screening Alfalfa Populations

There are three breeding populations that have been the focus of selection for CRC larval feeding resistance in the Cornell Forage Breeding Program (1) Oneida Ultra [21], (2) Seedway 9558 [22], and (3) a population derived from the eight plants of diverse background within the breeding program with the least amount of larval feeding in a 2000 space plant nursery (“Best 8”). 

Beginning in 2006, a phenotypic recurrent selection program was initiated with each cycle consisting of the following: For each half-sib in the three populations, forty seeds were seeded into vermiculite-filled greenhouse flats. At eight weeks, seedling roots were pruned to 10 cm and seedling tops were pruned to 12.5 cm. These seedlings were transplanted to a field nursery using an Ellis transplanter with 27 cm spacing between plants. Natural CRC populations were allowed to develop for three growing seasons and then in the fall, remaining plants were dug with a Egedal plant lifter, preserving as much root as possible. The 120 to 150 most vigorous plants from each population with the least CRC feeding were selected and brought into the greenhouse where they were interpollinated with bumble bees [*Bombus impatiens* Cresson (Hymenoptera: Apidae)]. Seed produced was used for planting the next cycle of selection. By the spring of 2014, three cycles of selection for resistance and one cycle for susceptibility to CRC had been completed.

In 2014, we established a field evaluation to determine gains in resistance due to selection. Populations consisting of equal numbers of seeds from each half-sib bulked together from each breeding line (Seedway 9558-3 cycles resistance, Seedway 9558-1 cycle susceptibility, Oneida Ultra-3 cycles resistance, Oneida Ultra-1 cycle susceptibility, and CRC resistant “Best 8”-2 cycles resistance). These breeding lines as well as the unselected base population seed from Seedway 9558 and Oneida Ultra were established into a field space plant nursery in a manner consistent with previous cycles of selection. In the fall of 2016, plants were dug, roots were washed clean with hoses, and each plant was given a CRC rating of 1 to 5 (1 = no feeding damage on roots, 5 = severe feeding damage). Plants with ratings of ones and twos were considered resistant and all others were considered susceptible. One additional cycle of selection was completed for each population for resistance or susceptibility concurrently with the evaluation to create the following populations, which were then sent to Utah State University for further testing (see Section 2.3):

Seedway 9558                    –4 cycles resistance → NY1713
                                            –2 cycles susceptibility → NY1718
Oneida Ultra                     –4 cycles resistance → NY1720
                                           –2 cycles susceptibility → NY1717
CRC resistant “best 8”     –3 cycles resistance → NY1719

### 2.2. Field Screening Data Analysis

CRC larval feeding damage ratings were analyzed using the R environment for statistical computing and tidyverse packages within RStudio [23]. Analysis of variance was performed and multiple comparisons using estimated marginal means were calculated with the emmeans package [24] for statistically significant fixed effects using the Tukey method for *p*-value adjustments. *p*-values of 0.05 or less were considered statistically significant.

### 2.3. Lab Screening CRC-Resistant Alfalfa Populations

Three developmental CRC-resistant (NY1713, NY1720, and NY1719) and two CRC-susceptible (NY1718 and NY1717) non-commercial populations from Cornell University were tested. Developmental populations were derived from Oneida Ultra (NY1720 and NY1717) or Seedway 9558 (NY1713 and NY1718) parental alfalfa populations. NY1719, which can be traced back to 8 plants from diverse backgrounds in the Cornell breeding program showing excellent vigor and no CRC-feeding in a 2000 space plant nursery, was also tested. To investigate CRC-resistance in these breeding populations, all five populations were tested against CRC larvae with the CRC-susceptible populations, NY1718 and NY1717, serving as positive controls. Further, the CRC-resistant population NY1713 paired with its CRC-susceptible population, NY1718, was tested against CRC adults.

### 2.4. Clover Root Curculio Collection for Assays

Clover root curculio eggs were collected from wild populations located at Greenville Research Farm (Logan, UT, USA) whereby a hand trowel was used to collect soil to a depth of 2.5 cm (~300 mL of soil per sample) around alfalfa crowns [1] during October of 2017 and 2018. Soil samples were processed similarly to the methods described by Rim et al. [1]. Briefly, soil was washed through a series of sieves (U.S. standard set #5, #10, #35, #60), particulate matter from the #60 sieve was examined under a stereomicroscope, and mature CRC eggs were placed on a moistened filter paper within a parafilm-sealed petri dish. Eggs were stored in the refrigerator (5–7 °C) until use in larval trials (one to four weeks after collection) when neonate larvae emerged from eggs (see Section 2.5).

Adult CRC were field collected from August to October of 2018 and 2019 using a handheld vacuum mulcher (Echo ES-250, Lake Zurich, IL, USA) modified into a sampling device. Clover root curculio adults recovered from vacuum samples were placed in a bug dorm (BioQuip Products Inc., Compton, California, USA) with a moistened cotton roll (Patterson Companies, Saint Paul, MN, USA) and alfalfa bouquets replenished every three to four days. Clover root curculio adults are not sexually dimorphic; therefore, we observed copulation or mate guarding behavior for sex determination and collected adult pairs in a 9-dram vial with a moist cotton wick. Adults were subsequently stored at 5–7 °C for one to two weeks and cotton wicks were moistened *ad libitum* until their use (see Section 2.6)

### 2.5. Larval Assays

**Figure 1:**
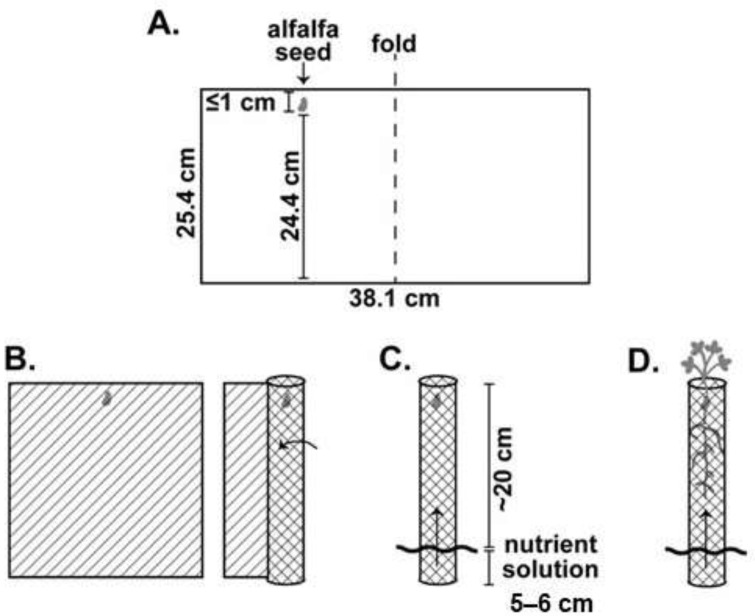
Alfalfa plants were grown hydroponically to be tested against CRC larvae. (**A**) One rhizobia-inoculated (Nitragin^®^ Gold, Monsanto, St. Louis, MO, USA) alfalfa seed was placed no more than 1 cm from the top edge of a moistened germination paper (38.1 × 25.4 cm) (Anchor Paper Inc, St. Paul, MN, USA), which was then folded in half. (**B**) Papers with seeds were rolled into cylinders, and (**C**) placed into containers filled with a hydroponic growth solution (0 N-10 P-10 K, Alaska Morbloom, Central Garden and Pet Company, Walnut Creek, CA, USA; Immunox Multi-purpose Fungicide, Spectracide, Spectrum Brands, Madison, WI, USA) covering the bottom 5–6 cm of papers. Capillary action through the papers appropriately moistened seeds for germination and growth. (**D**) Plants were grown for approximately 2–4 months prior to use in larval trials.

**Figure 2:**
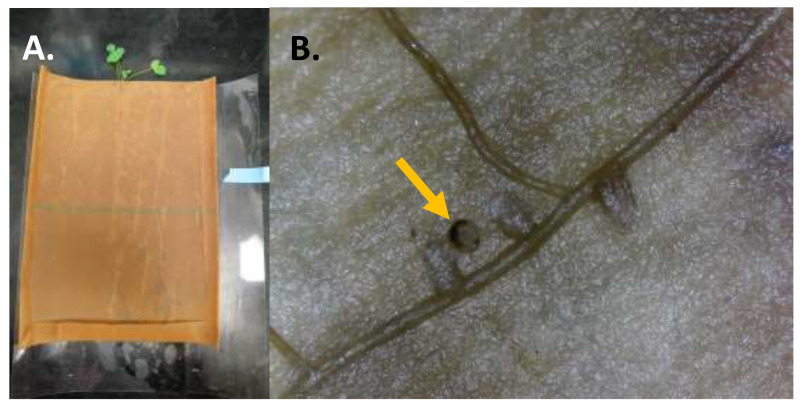
To evaluate alfalfa root nodule feeding and CRC larval survivorship and development, the arena (**A**) consisting of an outer transparency film cover and developing plant within germination paper was unfurled. The germination paper provided a substrate for roots and nodules to develop (**B**) and where a CRC larva (yellow arrow) could move and feed throughout the root zone, particularly on nodules.

Plants used in the larval study were grown hydroponically inside an incubator (#136LLVL Percival, Perry, IA) under constant environmental conditions (23–25 °C, 14L:10D, 40% RH) for two to four months until use (Figure 1A–D). To standardize root nodule numbers at the initiation of the larval feeding experiment, nodules were randomly excised using a knife (X-ACTO, Elmer’s Products Inc., High Point, NC, USA) so that only four to sixnodules remained at the start of the experiment. We used a completely random design to assign treatments (uninfested control or CRC infested) with one egg placed adjacent to the crown of each CRC infested plant (~1 cm deep). Uninfested controls were used to ensure no other factors were contributing to root material loss and/or growth during the experimental period. Each plant and egg, secured between germination paper, were rolled into transparency film for stability (Figure 1 and Figure 2A). We established 15 replicates of each alfalfa population (for both uninfested control and CRC infested) within each respective trial and held them in the same incubator conditions as described above for plants. Eggs were monitored every 24 h until first-instar larvae emerged, after which feeding continued on the plant undisturbed for one week. At the end of one week, surviving larvae were carefully removed, their head capsule width (mm) measured, and nodules observed for damage (Figure 2B). Data were also recorded for the number of additional nodules that developed during the experiment. 

### 2.6. Adult Assays 

Three to five plants were seeded in a 15.25 cm diameter × 20.32 cm tall pot (experimental unit) filled with Sungro #3 Propagation Mix under greenhouse conditions (23 °C, 14L:10D, 37% RH) until plants were approximately 30 cm in height. One of the germinated plants was randomly selected from each pot (20 replicates for each alfalfa population) to be used in the experiment and all others were removed. To standardize the number of leaves, leaves were removed until there were only five trifoliate leaves per plant. Transparent enclosures (cages) were constructed by rolling transparency film into a ~5 cm diameter by 27.94 cm-long tube with a rubber band secured mesh bag over one end (top of the cage). Plaster of Paris (DAP Products, Baltimore, MD, USA) mixed as directed was poured over the freshly watered soil, creating a flat, white surface to aid in CRC egg collection. The uncovered bottom of each cage was centered around each plant stem and pushed into the unset plaster, creating a plaster floor inside the cage. The plaster set for 30 min before CRC pairs were added. Plants were exposed to a mated pair for four days and beetle survival was recorded every 24 h throughout the study. Replications from adult evaluations where one or both beetles died or escaped prior to the four-day experimental period were excluded. Surviving beetles were then removed and placed in a 70% ethanol solution for sex confirmation via dissection, and eggs were counted within each cage. When CRC dissections revealed both beetles to be male, this replication was also removed from oviposition data. To estimate adult feeding damage (leaf area consumed and total leaf area), stems were cut at the base and shoot material was reserved. Additionally, negative controls were used to observe for indiscriminate oviposition behavior by placing CRC pairs into cages with a moistened cotton roll without plants. 

To estimate leaf consumption, first, freshly collected shoot material was carefully spread on a flatbed scanner to obtain a digital image. A ruler was included for scale. Using Photoshop (Adobe, San Jose, CA, USA), voids where feeding damage occurred were filled with a contrasting color and the remainder of plant material was removed from the photo so that only damaged area was shown. Similarly, total leaf area estimates were taken by tracing and filling damaged area in addition to remaining undamaged leaf material. These images were then analyzed with ImageJ (ImageJ 1.49f; http://rsbweb.nih.gov/ij/, (accessed on 4 October 2021)), calculating the total damaged area and surface area (mm^2^) for all five leaves combined.

### 2.7. Lab Screening CRC-Resistant Alfalfa Population Data Analysis

All data were analyzed using R software (RStudio). Developmental alfalfa populations were evaluated for resistance (NY1720, NY1713, and NY1719) and susceptibility (NY1717 and NY1718) to CRC larvae. Count data were analyzed using GLM with the log_10_-link and Poisson distribution error to compare the total number of nodules consumed on alfalfa populations, and the quasi-Poisson distribution error for the number of nodules grown between treatments (uninfested control and CRC-infested) for each plant population with an average of ≥1 grown nodules. To compare group means, Tukey tests (95% confidence index) for multiple comparisons were applied to GLMs using the MultComp package. Independent Kruskal-Wallis rank sum tests for non-normal data were used to analyze the proportion of nodules consumed ($\sin^{-1}\sqrt{\frac{\#\;nodules\;consumed}{Total\;\#\;nodules}}$) and larval head capsule width (mm) data. When the Kruskal–Wallis tests resulted in statistical significance (*p* ≤ 0.05) pairwise comparisons using the Wilcoxon rank sum test with Bonferroni correction were performed. 

Replications from adult evaluations where one or both beetles died prior to the four-day experimental period were excluded leaving 17 replications for each population tested (NY1718 and NY1713). To assess differences in leaf consumption between CRC-resistant and susceptible lines the proportion of leaf area consumed ($\sin^{-1}\sqrt{\frac{\;leaf\;area\;consumed\;({\mathrm{mm}}^{2})}{total\;leaf\;area\;({\mathrm{mm}}^{2})}}$) was analyzed by a one-way ANOVA. Tukey’s HSD post hoc tests followed ANOVAs to separate significant differences in adult feeding among lines. 

Dissections for one replication revealed two females, thus, the total number of eggs laid (oviposition) for this replication was divided by two to adjust to one gravid female. Oviposition during the experimental period (four days) was compared among CRC-susceptible population, NY1718, and CRC-resistant population, NY1713, using a GLM with the log_10_-link and quasi-Poisson distribution error. Following the GLM analysis, Tukey HSD with a 95% confidence index was performed using the MultComp package for multiple comparisons. 

## 3. Results

### 3.1. Field Screening Alfalfa Populations

**Figure 3:**
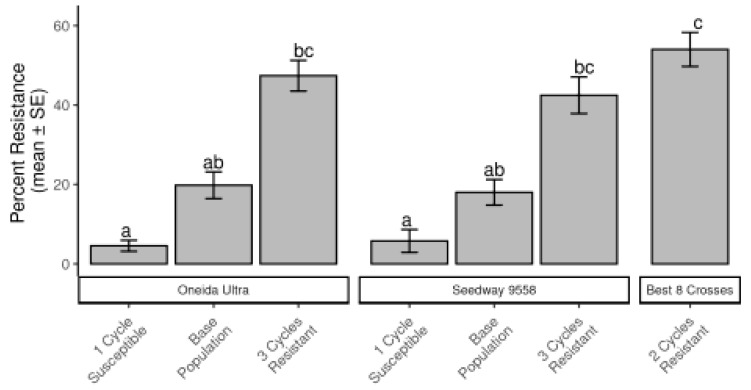
Comparison of alfalfa population mean (±SEM) percent resistance for populations resulting from divergent selection within cultivars Oneida Ultra and Seedway 9558, and a population developed from the best eight plants selected from a field and intercrossed for CRC resistance for two cycles of selection. Different letters above bars indicate significant differences resulting from Tukey HSD.

Over the two alfalfa cultivars, populations that had gone through selection for CRC resistance averaged 42% resistance, the original unselected cultivars averaged 18% resistance, and the susceptible selections averaged 6% resistance based on root damage ratings. The population developed from the CRC resistant “Best 8” had 56% resistance, but this percentage was not statistically greater than the other resistant populations (Figure 3).

### 3.2. Lab Screening CRC-Resistant Alfalfa Populations 

The total number of nodules consumed by larvae did not differ among developmental alfalfa populations (GLM: F = 1.447, *p* = 0.216) (Table 1). However, the proportion of nodules consumed on NY1713, the Seedway 9558-derived CRC-resistant population, was approximately 3 times less than NY1720, the Oneida Ultra-derived CRC-resistant population (Kruskal–Wallis: χ^2^ = 12.992, df = 4, *p* = 0.011) (Table 1). Only the NY1713 population grew more than one nodule on average (4.17 ± 1.12 nodules) over the seven-day experimental period compared to the other Cornell developmental populations (0.21 ± 0.12 nodules). Yet, NY1713 nodules grew similarly on the uninfested control (4.53 ± 1.94 nodules) and the CRC-infested treatment (3.8 ± 1.21 nodules) (GLM: F = 0.107, *p* = 0.747). Larval head capsule widths did not differ among the developmental populations (Kruskal-Wallis: χ^2^ = 7.051, df = 4, *p* = 0.133). Yet, the size range of head capsule widths for all alfalfa populations except NY1718 were effectively classified as 3rd instar, while NY1718 was 4th instar [24].

**Figure 4:**
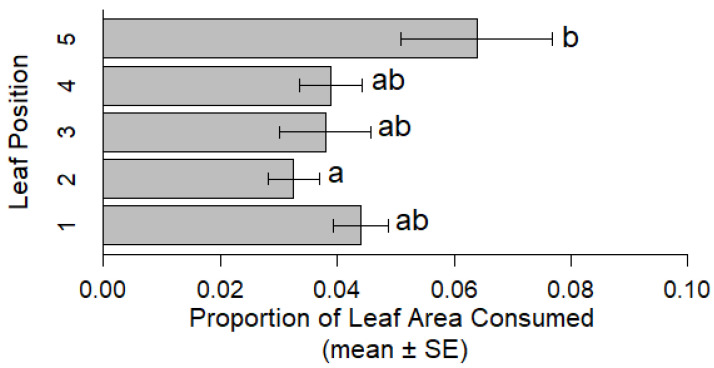
Comparison of leaf area consumed by adult CRC (NY1718 and NY1713 combined) by leaf position with 1 being the basal most trifoliate leaf and 5 the apical trifoliate leaf. Values shown are the mean (±SEM) proportion of leaf area consumed by caged mated pairs of adults. Different letters above bars indicate significant differences resulting from Tukey HSD.

In adult evaluations with CRC-resistant NY1713 and CRC-susceptible NY1718 populations, the proportion of leaf area consumed was similar among populations (ANOVA: F = 0.015, df = 1, 32, *p* = 0.912) (Table 2). The proportion of leaf area consumed by adults was significantly greater on the apical trifoliate leaf compared to the lower basal trifoliate leaves (Figure 4). Adults consumed nearly two times the leaf area on the uppermost trifoliate leaf compared to leaf position 2. When evaluating oviposition (total eggs), there was no significant difference between NY1718 and NY1713 (GLM: F = 0.032, *p* = 0.860) (Table 2).

## 4. Discussion

Clover root curculio represents an alfalfa insect with renewed and elevated pest status, thought to be a result of the ban of soil-active broad-spectrum insecticides in the alfalfa system [1]. CRC’s increase and limited management options including crop rotation and altering planting date have been less successful in reducing populations and resulting crop damage and yields. While alfalfa resistance to insects with piercing-sucking mouthparts (e.g., aphids) and for pathogens (e.g., *Fusarium* wilt and nematodes) exists [11], the development of resistant cultivars for chewing insects has been challenging. However it is progressing for root-feeding larvae and offers another management approach [20]. Through the Cornell Plant Breeding Project, recurrent phenotypic selection has improved resistance in three breeding populations over CRC-susceptible ones. Together, these results are in line with screenings that eventually led to the release of the alfalfa cultivar resistant to the alfalfa snout beetle, with similar pest characteristics, namely root larval feeding, found with CRC. It is important to note that NY1713 and NY1718 are derived from the Seedway 9558 parental line involved in alfalfa snout beetle resistant alfalfa [22]. Field screening of alfalfa for below-ground pests has some limitations, in part, because CRC is difficult to rear, and direct population manipulations are challenging to achieve, leaving screening to focus on resident populations that can have a patchy distribution. Nonetheless, extensive screening over long periods of time in our research has shown distinct patterns of alfalfa resistance to CRC. Considering the rating system offered by the National Alfalfa and Forage Alliance for alfalfa cultivar ratings, the screenings here represent the upper resistance classes of resistance (31–50% resistant plants) and high resistance (>50% resistant plants) [11]. 

Considering the challenges for field screening, we developed a soil-less method to closely evaluate root-feeding by CRC. Here, we could focus on the early instar larvae to investigate root severing and nodule feeding, particularly since egg collection is more easily attainable than larvae. Understanding the potential mechanisms behind resistance to CRC in the Cornell University developmental alfalfa populations is critical and may inform future advances in screening of belowground insects, particularly host plant resistance of chewing-insects. CRC-resistant alfalfa populations selected from field screening did not reduce larval feeding as larvae consumed a similar number of nodules on the CRC-susceptible and CRC-resistant developmental alfalfa populations. While larval development also appeared to be similar on CRC-susceptible and CRC-resistant alfalfa, head capsule widths for NY1718, a CRC-susceptible alfalfa, were categorized as 4th instar larvae [25]. All other developmental alfalfa, namely the CRC-resistant alfalfa (NY1713 and NY1719) had head-capsule ranges primarily within the 3rd instar larva category [25]. This suggests a possible reduction in development time with NY1713 and NY1719 and a need for additional investigation on mechanisms related to a delay in development. Considering these weak effects, experimental methods herein did differ from those of our field screening and Crawford et al. [20]. Considering the challenges of working with CRC previously described, field screening relies on rating overall taproot damage in the field over two years. We were able to supplement this work in a soil-less system for seven days. The soil-less arenas in our study allowed for easy and simplified observations of plant-herbivore interactions in belowground systems. This does not reflect the myriad of biotic and abiotic factors contributing to herbivore feeding, growth, and survival under field settings. For example, Hackell and Gerard [26] hypothesized that the inability of *Sitona lepidus* Gyll. (Coleoptera: Curculionidae) larvae to feed on clover nodules in petri dishes was due to nodule odors flooding the experimental arena and a lack of contact stimuli. Contrastingly, in a field system, nodule odors are dispersed in the soil around nodules in gradients, and the larval cuticle is in contact with the soil medium [26]. Moreover, field screening offered an inherent choice of resident CRC selection among the alfalfa populations over time, a choice not provided in lab screening. The methods we employed in the larval experiments were sufficient for rapid screening of potentially resistant alfalfa populations, but further research in systems with soil are important to a well-rounded understanding of CRC larval feeding, development, and survival.

Although we did not observe differences in the total number of nodules consumed, the proportion of nodules consumed were lower on CRC-resistant alfalfa NY1713 compared to NY1720, also a CRC-resistant alfalfa. The significantly lower proportion of nodules consumed by larvae on NY1713 was because this line grew nodules during the seven-day experimental period. We hypothesized this may have been a compensatory response to larval feeding as evidenced by Quinn and Hall [27], where compensatory growth of alfalfa nodules occurred after nodules were removed by CRC or when mechanically removed. However, NY1713 nodule growth was similar between the uninfested control and the CRC-infested treatment. This suggests compensatory nodule growth did not occur in this CRC-resistant alfalfa, but that NY1713 tended to have increased nodulation overall. 

Enhanced nodulation may benefit host plants damaged by CRC by decreasing nitrogen stress and potentially increasing resiliency to CRC larval feeding. The negative impacts of larval feeding on nitrogen-fixing nodules and subsequent plant stress is well-known and has been recorded for other sitonids. For example, high populations of *S. lepidus* larvae terminated the nitrogen-fixing abilities of white clover plants [28], and *Sitona discoideus* Gyll. larval feeding disrupted nitrogen-fixation and increased nitrogen stress in field-grown alfalfa [29]. These decreases in nitrogen-fixation resulted in reduced aboveground nitrogen, yield, and decreased stem regrowth after harvest [29,30]. Further, Vankosky et al. [31] found that although the number of *S. lineatus* damaged nodules did not change in inoculant and thiamethoxam treated field pea, plants displayed increased nodulation, higher numbers of large multilobed nodules, and increased nitrogen fixation compared to controls. If CRC larvae consume the same number of nodules on average (~2 nodules/larva/week), the observed increased nodulation in N1713 may offset the nitrogen stress and disruption of nitrogen-fixation due to regular nodule loss from CRC larval feeding. However, further studies on nitrogen-fixation capacity related to CRC larval feeding on NY1713 is needed to test this hypothesis.

A possibility is that the increased nodulation observed on NY1713 may potentially increase CRC larval survival and may not necessarily result in subsequent plant yield increases. Although CRC and most *Sitona* spp. early instars are not obligate nodule feeders, research indicates that just the presence of nodules on alfalfa roots increases larval survival by 3–14 times when compared to larval survival on non-nodulated roots [14,32]. Further, CRC and *Sitona* spp. larval development (head capsule width and body length) was enhanced on nodulated roots [14,26,28,32]. Although NY1713 had a higher number of nodules, we did not observe increased head capsule widths for CRC larvae on NY1713 compared to the other developmental alfalfa. The greatest head capsule widths instead occurred with NY1718, the CRC-susceptible alfalfa. Additionally, *S. lepidus* larvae are attracted to nodules by the volatile protein asparagine [33], a precursor and potential host-finding and feeding stimulant for larvae [26]. Increased nodulation may increase volatile emission and, thus, increase host-plant or nodule finding. Lastly, first-instar *Sitona* mortality was high (95–99%) [34] and was posited to be a result of interspecific competition or failed host-plant and nodule finding [35]. While the increased nodulation observed on NY1713 may potentially increase host-plant and nodule-finding, the difference in instar developmental rates between CRC-resistant NY1713 (3rd instar) and CRC-susceptible NY1718 (4th instar) and resistance witnessed in the field screening suggests CRC may not be successful on NY1713 under field conditions. 

Aboveground adult CRC feeding did not differ between the CRC-resistant alfalfa, NY1713, and its paired susceptible line, NY1718, suggesting that purported resistance and susceptibility of these alfalfa populations to field populations of CRC larvae may not necessarily affect aboveground tissues and CRC adult feeding. When developing alfalfa cultivars with resistance towards chewing insect pests, it is important to increase resistance without sacrificing nutrition and palatability for livestock feeding on foliage. For example, high saponin content in alfalfa was associated with increased CRC resistance [36], but saponins have negative effects on animal metabolism and are associated with bloat (increased gas pressure in the rumen) in livestock [37]. For these reasons, developing alfalfa with resistance to CRC may be particularly difficult. Therefore, it could be beneficial that resistance or tolerance for these CRC-resistant alfalfa populations is concentrated in belowground tissues. Oviposition behavior was also similar between NY1713 and NY1718. Maternal CRC may be able to detect that belowground resources exist (nodules); however, female CRC in this study may not have had the ability to detect the increased number of nodules found on NY1713. Johnson et al. [38] posited that maternal congener *S. lepidus* received cues through host shoot and root volatiles as well as chemical and physical cues from the soil. We hypothesized that if female CRC were able to adjust oviposition based on the concentration of belowground resources (higher numbers of nodules), this ability may have been hindered in the current study since plaster covered the soil surface and may have disrupted detection of root volatiles and soil cues. 

Overall, CRC larvae were able to feed on nodules and develop on all alfalfa populations tested. Further, antibiosis was not observed with larvae or adults on CRC-resistant developmental populations. CRC-resistant developmental populations are continuously under development at the Cornell Plant Breeding Project, thus, upcoming breeding cycles of these populations may yield different or stronger results. Future studies should focus on evaluating alfalfa cultivars with resistance to other pests as well as investigate the interactions between enhanced nodulation, CRC larval survival, and nitrogen stress to determine if nodule growth is a beneficial physiological adaptation that increases alfalfa resilience towards CRC larvae. Lastly, bioassay procedures to observe belowground insect pests are lacking [39]. Therefore, the method described here to observe larvae is a novel way to screen host plant cultivars and observe belowground plant-herbivore interactions without expensive equipment or the extensive time commitment that usually accompanies soil sorting methods.

## 5. Conclusions

The lack of management options and the cryptic nature of CRC larvae (the damaging life stage) has led to the elevation of this pests’ status within the alfalfa system. Field screenings of alfalfa populations from the Cornell Forage Breeding Program provide several candidate alfalfa populations with resistance to CRC. These alfalfa populations result from three breeding populations where resistance has improved with increasing cycles of selection. Although the mechanisms of pest resistance in alfalfa populations are difficult to pinpoint, development of soilless lab screening protocols offer a way to evaluate root-larva interactions. Through this approach, observations on nodule development, nodule consumption, and instar begin to highlight particular CRC-resistant alfalfa populations (i.e., NY1713) to investigate further. Considering the success in developing resistant alfalfa to alfalfa snout beetle, the possibility for developing a host-plant resistance management tool for CRC to assist alfalfa producers appears promising.

## Figures and Tables

**Table 1:** Larval CRC feeding and development on each of five developmental alfalfa populations. Mean (±SEM) total number of nodules, number of nodules consumed by larval CRC, proportion of nodules consumed, and larval CRC head capsule width for each alfalfa populations. Different letters within column indicate significantly different means via Kruskall-Wallis rank sum test (*p* ≤ 0.05).

Line	Resistance/ Susceptibility	No. Nodules	No. Nodules Consumed	Proportion of Nodules Consumed	Larval Head Capsule Width (mm)
NY1718	CRC-susceptible (2 cycles)	4.33 ± 0.33	2.33 ± 0.88	0.57 ± 0.23 ab	0.59 ± 0.05
NY1717	CRC-susceptible (2 cycles)	4.67 ± 0.43	2.17 ± 0.54	0.44 ± 0.09 ab	0.50 ± 0.04
NY1713	CRC-resistant (4 cycles)	9.20 ± 1.32	1.53 ± 0.17	0.22 ± 0.04 a	0.43 ± 0.03
NY1720	CRC-resistant (4 cycles)	5.11 ± 0.39	3.00 ± 0.41	0.64 ± 0.11 b	0.50 ± 0.05
NY1719	CRC-resistant (3 cycles)	5.40 ± 0.58	2.30 ± 0.42	0.48 ± 0.10 ab	0.43 ± 0.04

**Table 2:** Mean (±SEM) total leaf area, area consumed, proportion of area consumed, and oviposition by adult CRC on CRC-susceptible (NY1718) and CRC-resistant (NY1713) developmental populations. Proportion of leaf area consumed was calculated by dividing leaf area consumed by total leaf area.

Line	Total Leaf Area (mm^2^)	Leaf Area Consumed (mm^2^)	Proportion of Leaf Area Consumed	Oviposition
NY1718	1774.58 ± 140.57	67.67 ± 7.78	0.04 ± 0.01	22.53 ± 5.11
NY1713	2198. 80 ± 175.11	78.65 ± 9.78	0.04 ± 0.01	23.76 ± 4.67
